# Spliceosomal components protect embryonic neurons from R-loop-mediated DNA damage and apoptosis

**DOI:** 10.1242/dmm.031583

**Published:** 2018-02-01

**Authors:** Shelly Sorrells, Sara Nik, Mattie Casey, Rosannah C. Cameron, Harold Truong, Cristhian Toruno, Michelle Gulfo, Albert Lowe, Cicely Jette, Rodney A. Stewart, Teresa V. Bowman

**Affiliations:** 1 Department of Oncological Sciences, Huntsman Cancer Institute, University of Utah, Salt Lake City, UT 84112, USA; 2 Department of Developmental & Molecular Biology, Albert Einstein College of Medicine, Bronx, NY 10461, USA; 3 Gottesman Institute for Stem Cell Biology and Regenerative Medicine, Albert Einstein College of Medicine, Bronx, NY 10461, USA; 4 Department of Medicine (Oncology), Albert Einstein College of Medicine, Bronx, NY 10461, USA

**Keywords:** Splicing, Radiation, Apoptosis, Zebrafish, R-loops, Neurons

## Abstract

RNA splicing factors are essential for the viability of all eukaryotic cells; however, in metazoans some cell types are exquisitely sensitive to disruption of splicing factors. Neuronal cells represent one such cell type, and defects in RNA splicing factors can lead to neurodegenerative diseases. The basis for this tissue selectivity is not well understood owing to difficulties in analyzing the consequences of splicing factor defects in whole-animal systems. Here, we use zebrafish mutants to show that loss of spliceosomal components, including *splicing factor 3b, subunit 1* (*sf3b1*), causes increased DNA double-strand breaks and apoptosis in embryonic neurons. Moreover, these mutants show a concomitant accumulation of R-loops, which are non-canonical nucleic acid structures that promote genomic instability. Dampening R-loop formation by conditional induction of ribonuclease H1 in *sf3b1* mutants reduced neuronal DNA damage and apoptosis. These findings show that splicing factor dysfunction leads to R-loop accumulation and DNA damage that sensitizes embryonic neurons to apoptosis. Our results suggest that diseases associated with splicing factor mutations could be susceptible to treatments that modulate R-loop levels.

## Abstract (precis)

**Summary:** Loss of RNA splicing factors causes R-loop accumulation and DNA damage in embryonic neurons, sensitizing them to radiation-induced cell death. These findings suggest that diseased cells with mutations in splicing factors are vulnerable to radiotherapy.

## INTRODUCTION

R-loops are nucleic acid structures that have crucial roles in several cellular processes, including mitochondrial DNA replication, immunoglobulin class-switch recombination and regulation of transcription (Santos-Pereira and Aguilera, 2015). An R-loop is a three-stranded nucleic acid structure comprised of an RNA:DNA hybrid and the associated non-template single-stranded DNA; they can form when a nascent pre-mRNA transcript binds to the complementary strand of DNA (Santos-Pereira and Aguilera, 2015). Although the *in vivo* role of R-loops is poorly understood, links between the aberrant accumulation of R-loops and several human diseases, such as the neuro-inflammatory disease Aicardi-Goutières Syndrome, nucleotide expansion diseases and cancer, suggest that proper regulation of R-loop levels is important for tissue homeostasis (Groh and Gromak, 2014).

Splicing factors are best known for their role in removing non-coding, intronic sequences from protein-coding RNA transcripts during mRNA maturation (Will and Lührmann, 2011). Splicing factors can also regulate R-loop dynamics (Santos-Pereira and Aguilera, 2015), as impairing splicing factor function can cause accumulation of R-loops that eventually leads to the formation of DNA double-stranded breaks (DSBs) (Paulsen et al., 2009). Thus, although mutations in splicing factors are known to be prevalent in many human diseases, such as neurodegeneration, it is not known if disruption of pre-mRNA splicing or other non-canonical roles of splicing factors drive disease (Dvinge et al., 2016; Szafranski et al., 2015). A barrier to understanding how splicing factor mutations contribute to disease is an incomplete knowledge of the tissue- or cell-type-specific roles of these factors in animal models. This is due, in part, to the embryonic lethality associated with homozygous loss of canonical splicing factors in mammalian systems.

To identify novel genes that normally protect embryonic tissue from ionizing radiation (IR)-induced apoptosis, we performed a forward genetic screen in zebrafish and found that loss of the splicing component *coiled-coil domain containing 94* (*ccdc94*) caused neural-specific sensitivity to IR before any major defects in mRNA splicing were observed (Sorrells et al., 2012). Analysis of *ccdc94* mutants showed that levels of tumor protein p53 (Tp53) and *tp53* mRNA were significantly elevated in these mutants, leading us to hypothesize that increased Tp53 levels were causing the increased sensitivity to IR-induced apoptosis. By evaluating both splicing and non-splicing factor mutants with different levels of Tp53, we found to our surprise that sensitivity to IR-induced neuronal apoptosis did not correlate with Tp53 levels, yet all splicing mutants were still radiosensitive. These data suggest that a non-canonical role of splicing factors might underlie the IR-induced neuronal apoptosis, and here we show that deregulation of R-loop physiology contributes to this phenotype. We show that in the absence of IR, several splicing factor mutants exhibit accumulation of the DNA DSB marker γH2AX, which is independent of Tp53. We demonstrate that R-loop levels are elevated in mutants for the spliceosomal component Sf3b1 (Splicing factor 3b, subunit 1), especially in neurons, and that depletion of R-loops via conditional expression of ribonuclease H1 alleviates the increase in DNA DSBs and apoptosis in *sf3b1* mutants. Our data suggest that embryonic neural tissue is exquisitely sensitive to R-loop-mediated genomic instability from splicing factor deficiency and that this trait could enhance the therapeutic index of IR treatment for diseases with dysfunctional mRNA splicing, particularly those arising from embryonal neural precursor cells.

## RESULTS

### Disruption of RNA splicing factor genes sensitizes zebrafish embryonic neural tissue to IR-induced apoptosis

**Fig. 1.**
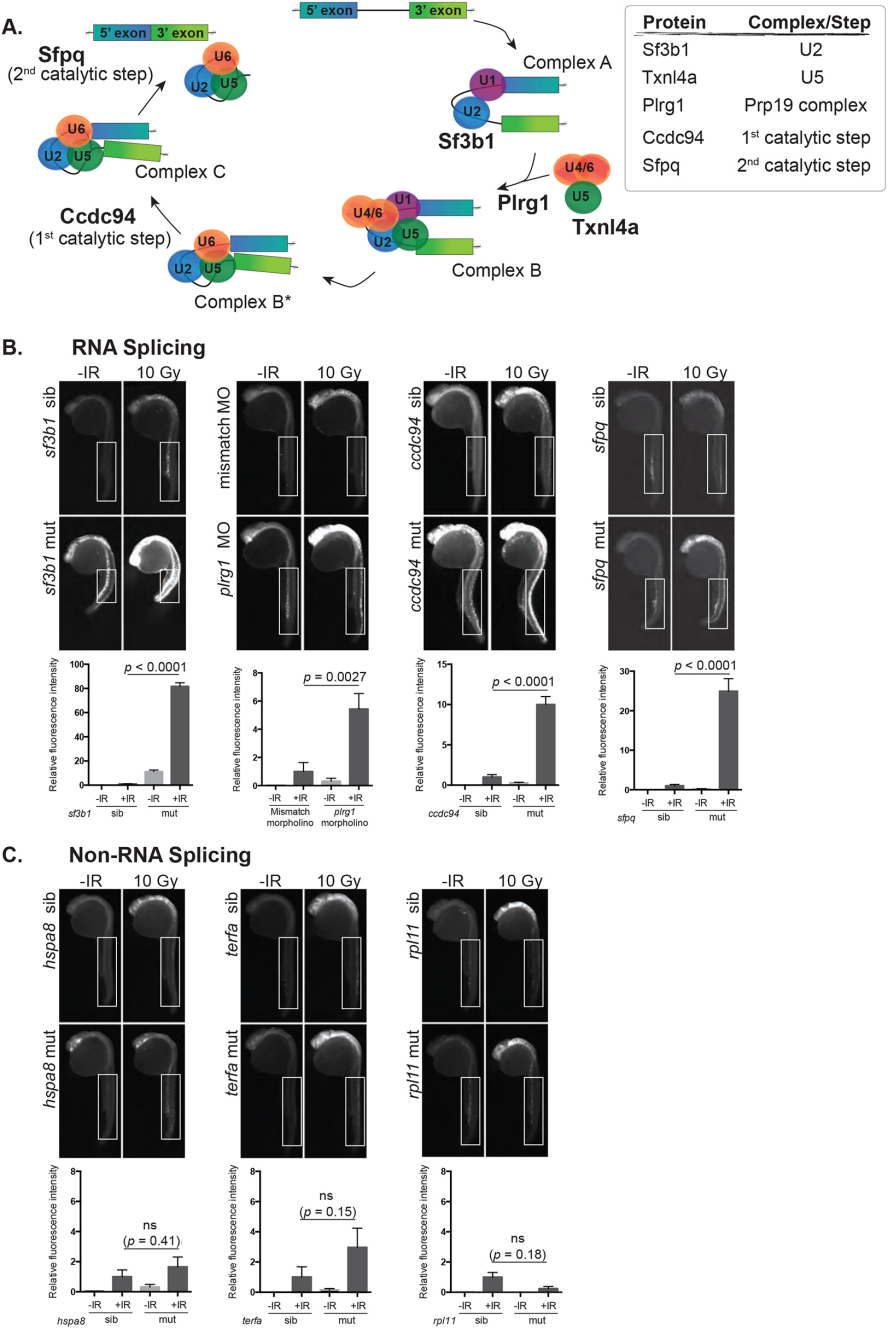
**Disruption of RNA splicing factor genes sensitizes embryonic neurons to IR-induced apoptosis.** (A) Schematic of the RNA splicing pathway showing the steps in which the RNA splicing factors used in this study are expected to function. (B) Splicing factor mutant and sibling embryos, or morphants and controls, were exposed (+IR) or left unexposed (-IR) to 10 Gy IR and analyzed 3 h later for active caspase-3. All embryos were irradiated at 24 hpf, except *sf3b1^hi3394aTg^* mutants and siblings which were irradiated at 22 hpf before the neurodegenerative phenotype became severe. Upper panels show representative images of active caspase-3 staining in each genotype or treatment group. Lower panels show the quantification of active caspase-3 staining for each genotype measured in the spinal cord area within the boxed regions. At least 10 embryos were quantified per genotype and treatment condition and fluorescence intensity from sibling +IR was normalized to one to account for non-specific background staining. (C) Non-splicing factor mutants and siblings were analyzed as in B. ns, not significant; error bars represent s.e.m.

A recessive F3 ethylnitrosurea-based mutagenesis screen was previously described, in which zebrafish embryos with recessive radiosensitizing mutations were identified (Sorrells et al., 2012). The screen was performed by exposing 24 hour post-fertilization (hpf) embryos from the F3 generation with sub-threshold levels of IR and analyzing them 3-6 h later to identify mutants that have increased accumulation of cell death in the head, thus representing novel radiosensitizing mutations. The first mutant to be analyzed from the IR sensitivity screen (*ccdc94^zd1000^*), demonstrated a neuron-specific radioprotective function for the RNA splicing factor *ccdc94* (known as *YJU2* in yeast) (Sorrells et al., 2012). A component of the U5 RNA splicing complex, *thioredoxin-like 4a* [*txnl4a*, also known as *defective entry into mitosis 1* (*dim1*)] (Gottschalk et al., 1999), was the affected gene in the second mutant (*txnl4a^zd1006^*) to be identified (Fig. S1). The identification of two splicing factors in this unbiased genetic screen, coupled with earlier findings that loss of the splicing factors Pre-mRNA processing factor 19 (Prp19) and Pleiotropic regulator (Plrg1) also caused sensitivity to IR-induced apoptosis in embryonic neurons (Sorrells et al., 2012), led us to hypothesize that survival of embryonic neurons is selectively sensitive to general disruption of RNA splicing. To test this hypothesis, additional genes were analyzed at different steps of the canonical RNA splicing pathway (Fig. 1A) to determine whether this pathway was required to protect embryonic neural tissues from IR-induced apoptosis. In addition to *ccdc94*, *txnl4a* and *plrg1*, we chose *sf3b1* and *splicing factor, proline and glutamine rich* (*sfpq*) (Shav-Tal and Zipori, 2002; Will and Lührmann, 2011) for this analysis on the basis of the availability of previously established mutant lines (*sf3b1^hi3394aTg^*, *sfpq^hi1779Tg^*) (Amsterdam et al., 2004) and their established roles at different steps of the splicing pathway (Fig. 1A). To determine whether the splicing factor mutants were radiosensitive, we incrossed heterozygous mutant animals, exposed (or left unexposed) their 24 hpf progeny to 10 Gy IR and analyzed them 3 h later for active caspase-3 levels. The *ccdc94^zd1000^* mutants and *plrg1* morphants served as positive controls (Fig. 1B) (Sorrells et al., 2012). The *plrg1^hi3174aTg^* mutants exhibited a high level of neurodegeneration at 24 hpf that interfered with the analysis of radiosensitivity (Fig. S2A); thus, a low dose of the *plrg1* morpholino was used instead, which gave rise to obvious radiosensitivity with minimal neurodegeneration in the absence of IR (Fig. 1B) (Kleinridders et al., 2009; Sorrells et al., 2012). Similar to the *ccdc94^zd1000^* and *txnl4a^zd1006^* mutants (Fig. 1B and Fig. S1B, respectively) and the *plrg1* morphants (Fig. 1B), *sf3b1^hi3394aTg^* and *sfpq^hi1779Tg^* mutants show significant radiosensitivity (Fig. 1B). These data support our hypothesis that the splicing machinery is required for survival of embryonic neural tissue.

### Loss of essential housekeeping genes, per se, does not radiosensitize embryonic zebrafish neural tissue

The splicing factor mutants analyzed in this study are all recessive embryonic lethal, which led us to question whether radiosensitivity is simply a consequence of disrupting general cell-essential processes, or so-called ‘housekeeping’ genes, rather than a specific defect in RNA splicing. To address this possibility, we analyzed three zebrafish lines that harbor mutations in essential housekeeping genes that are not known to affect RNA splicing: the heat shock chaperone *heat shock protein family a, member 8* (*hspa8*, mutant allele *hspa8^hi138Tg^*), the telomere maintenance factor *telomeric repeat binding factor a* (*terfa*, mutant allele *terfa^hi3678Tg^*) and the 60S ribosomal component *ribosome protein l11* (*rpl11*, mutant allele *rpl11^hi3820bTg^*) (Amsterdam et al., 2004)*.* To determine whether disruption of *hspa8*, *terfa* or *rpl11* radiosensitizes zebrafish embryos, we incrossed heterozygous mutant animals, exposed (or left unexposed) their 24 hpf progeny to 10 Gy IR and analyzed them 3 h later for active caspase-3 levels. None of the mutants showed a significant increase in IR-induced apoptosis compared with siblings (Fig. 1C). These findings support a model in which disruption of RNA splicing factor genes, but not housekeeping genes in general, sensitizes zebrafish embryonic neural tissues to IR-induced cell death.

### Increased Tp53 protein is neither necessary nor sufficient to radiosensitize zebrafish embryonic neuronal tissue

**Fig. 2.**
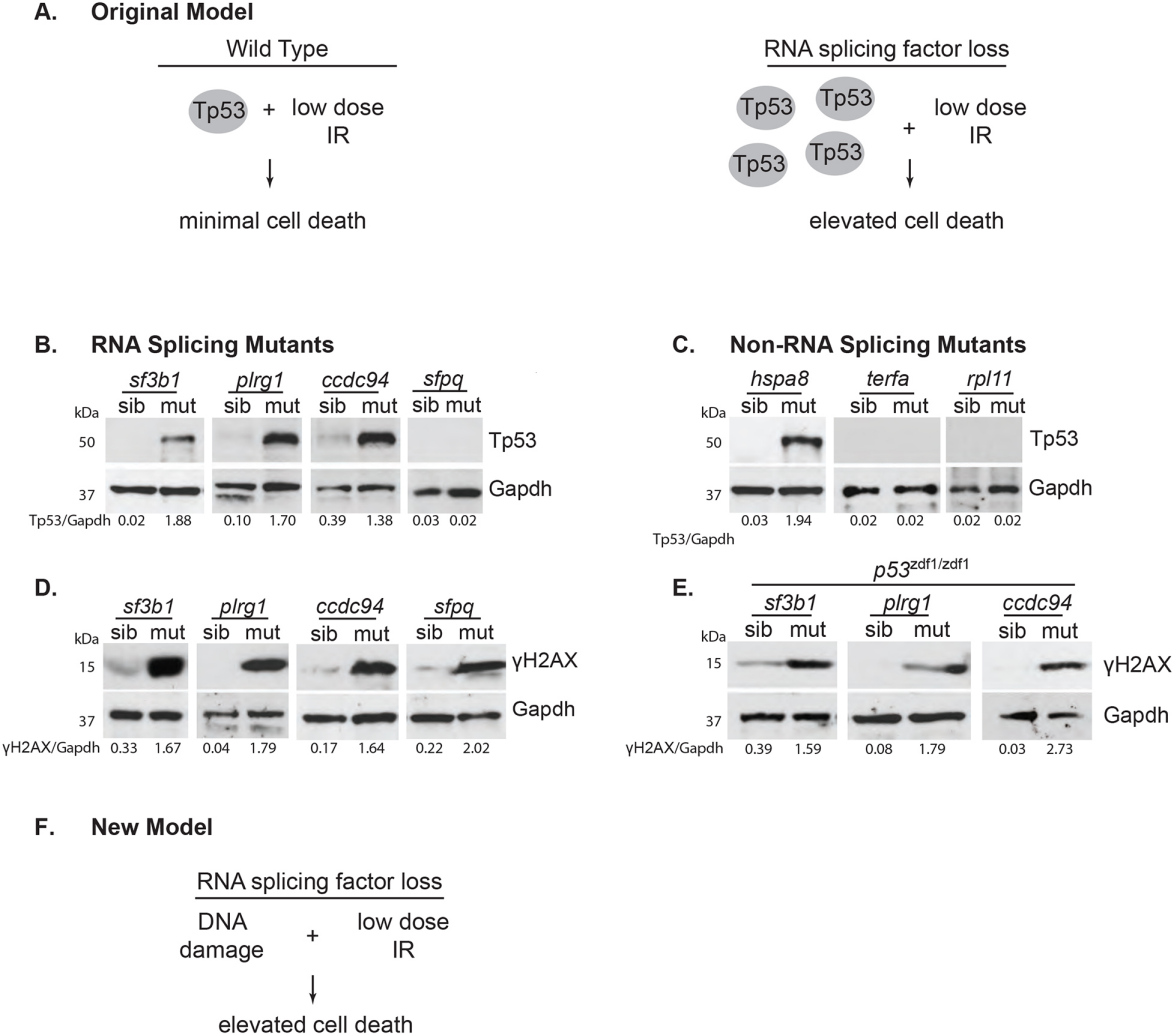
**Splicing factor mutants accumulate DNA DSBs in a Tp53-independent manner.** (A) Model of elevated Tp53 involvement in splicing factor mutant IR sensitivity. (B) Splicing factor mutants and siblings were separated by phenotype at 30 hpf and protein analyzed by western blot for Tp53 and glyceraldehyde-3-phosphate dehydrogenase (Gapdh, as a loading control). (C) Non-splicing factor mutants and siblings were analyzed as in B, but were genotyped (owing to a lack of overt phenotypes at this time point) before pooling for protein lysis. (D,E) Splicing factor mutants and siblings with the indicated genotype were separated at 30 hpf. Protein was analyzed by western blot for γH2AX and Gapdh. Relative levels of Tp53 or γH2AX normalized to Gapdh are shown below each western blot in B-E. Protein markers in kDa are shown to the left of each blot. (F) New model of DNA damage involvement in splicing factor mutant IR sensitivity.

Previous analysis of global mRNA splicing and transcript levels in *ccdc94^zd1000^* mutants by RNA sequencing showed that splicing was largely unaffected at the developmental stage (30 hpf) when embryos displayed sensitivity to IR-induced apoptosis (Sorrells et al., 2012). In addition, transcript levels were largely normal in *ccdc94^zd1000^* mutants with the exception of *tp53* mRNA and protein levels, which were significantly elevated. These findings led us to hypothesize that increased Tp53 levels were causing the increased sensitivity to IR-induced apoptosis. In support of this hypothesis, *plrg1^hi3174aTg^* mutants also showed increased levels of *tp53* pre-mRNA, spliced mRNA and protein expression, and loss of *tp53* completely abrogated the IR-induced apoptosis in wild-type and mutant embryos. Because Tp53 can both sense IR-induced DNA damage and execute apoptosis, the question remained as to whether increased Tp53 levels in *ccdc94^zd1000^* mutants were driving the IR sensitivity or simply acting in the final stages of the apoptotic process initiated by another upstream trigger. That is, elevated Tp53 levels might heighten the detection of IR-induced DNA damage at sub-threshold levels (Fig. 2A), which in turn triggers post-translational modification of the Tp53 protein to induce apoptosis (Dai and Gu, 2010). If this model is correct, we reasoned that an increase in Tp53 protein expression would be necessary and sufficient to radiosensitize zebrafish embryos. To uncouple Tp53 function in sensing DNA damage and promoting apoptosis, and hence test our model, we first analyzed Tp53 protein levels in unirradiated siblings and mutants at 30 hpf. The abnormal development of *sf3b1^hi3394aTg^*, *plrg1^hi3174aTg^*, *ccdc94^zd1000^* and *sfpq^hi1779Tg^* mutants (Fig. S2A) allowed us to separate siblings and mutants with certainty for further analysis, although the lack of both an obvious morphological phenotype in 30 hpf *txnl4a^zd1006^* mutants (Fig. S1A, -IR) and a straightforward genotyping protocol precluded confident sorting of mutants and siblings for these and subsequent analyses. The *sf3b1^hi3394aTg^* mutants showed an increase in Tp53 protein and mRNA expression similar to that of the *plrg1^hi3174aTg^* and *ccdc94^zd1000^* mutants (Fig. 2B and Fig. S2B-D). Surprisingly, however, *sfpq^hi1779Tg^* mutants did not exhibit increased Tp53 protein and mRNA expression levels (Fig. 2B and Fig. S2B-D). These data show that the increased radiosensitivity in *sfpq^hi1779Tg^* mutants is not simply a consequence of increased Tp53 levels and, instead, other mechanism(s) are required to drive sensitivity to IR-induced cell death.

The possibility that increased Tp53 levels, through increased sensor activity, is still sufficient to confer a radiosensitive phenotype was investigated. To test this possibility, we initially tried to overexpress *tp53* mRNA in whole embryos; however, this proved toxic to wild-type zebrafish embryos probably because of its ability to directly drive apoptosis (data not shown). To overcome this problem, we used zebrafish *hspa8^hi138Tg^*, *terfa^hi3678Tg^* and *rpl11^hi3820bTg^* mutants that had been shown previously to have upregulated *tp53* mRNA relative to wild-type embryos at 48 hpf (Danilova et al., 2010, 2011). The levels of Tp53 protein were not analyzed in these previous studies, so we performed a western blot analysis of Tp53 in 30 hpf siblings and mutants from each of these lines. This analysis showed that *hspa8^hi138Tg^* mutants have elevated Tp53 protein levels comparable with *sf3b1^hi3394aTg^*, *plrg1^hi3174aTg^* and *ccdc94^zd1000^* mutants, whereas *terfa^hi3678Tg^* and *rpl11^hi3820bTg^* mutants do not (Fig. 2B,C). Because embryonic neurons in *hspa8^hi138Tg^* mutants are not radiosensitive (Fig. 1C), these data show that increased Tp53 protein levels alone are not sufficient to drive the sensitivity of embryonic neurons to IR-induced cell death. To confirm that the Tp53 pathway was active in *hspa8^hi138Tg^* mutants, we performed whole-mount *in situ* hybridization for *p53 upregulator of apoptosis* (*puma*), the Tp53 target gene that is largely responsible for executing apoptosis (Jette et al., 2008). Consistent with the results from western blots, *ccdc94^zd1000^* and *hspa8^hi138Tg^* showed elevated *puma* expression in the brain, whereas *sfpq^hi1779Tg^* mutants showed no obvious *puma* expression (Fig. S2E). Thus, although endogenous Tp53 is required for executing apoptosis in splicing factor mutants, elevated levels of Tp53 protein are not necessary (e.g. *sfpq^hi1779Tg^* mutants) or sufficient (e.g. *hspa8^hi138Tg^* mutants) for driving the selective sensitivity of developing embryonic neural tissue to IR-induced apoptosis.

### Zebrafish RNA splicing factor mutants have an increase in DNA DSBs that is independent of Tp53-mediated apoptosis

To determine the mechanism(s) driving neuronal sensitivity to IR-induced apoptosis in splicing factor mutants, the role of cellular processes regulated by splicing factors was examined. An obvious candidate was defects in mRNA splicing or alternative splicing that led to the formation of pro-apoptotic gene products. However, when we previously performed RNA sequencing in *ccdc94^zd1000^* mutants at 30 hpf, more than 96% of genes showed no significant differences (i.e. *P*>0.05) in mRNA expression and there was no evidence of changes to alternative splicing (Sorrells et al., 2012). This is probably due to perdurance of maternal *ccdc94* mRNA that was detected in the sequencing experiment. Thus, major defects in splicing in *ccdc94^zd1000^* mutants occur at a later time point in development after radiosensitization phenotypes are observed. These defects probably contribute to the eventual embryonic lethality in these mutants, even when apoptosis is rescued by *tp53* loss (data not shown).

Another established, and more immediate, response to splicing factor dysfunction is the aberrant accumulation of R-loops and the formation of DNA DSBs (Santos-Pereira and Aguilera, 2015). To investigate this response, we first analyzed γH2AX protein levels (a marker for DNA DSBs) (Rogakou et al., 1998) and found all splicing mutants had elevated levels of γH2AX protein compared with siblings (Fig. 2D). Notably, this analysis measures the developmental accumulation of DNA DSBs, as it was performed in the absence of IR. Therefore, to rule out the possibility that the increased γH2AX protein levels were simply a consequence of the low level of neuronal apoptosis commonly observed in these mutants (Fig. 1B and Fig. S2) (Kleinridders et al., 2009; Sorrells et al., 2012; De La Garza et al., 2016), we prevented apoptosis by generating double mutants between *tp53* and *sf3b1^hi3394aTg^*, *plrg1^hi3174aTg^*, and *ccdc94^zd1000^* (Berghmans et al., 2005). Analysis of γH2AX protein levels in these double mutants showed that inhibiting developmental apoptosis in splicing factor mutants through *tp53* deficiency does not prevent the accumulation of γH2AX (Fig. 2E,F). Thus, an early event in splicing factor dysfunction is the accumulation of DNA DSBs, which occurs independently of Tp53 function and developmental apoptosis.

### Embryonic cells from *sf3b1* mutants show an increase in R-loops

**Fig. 3.**
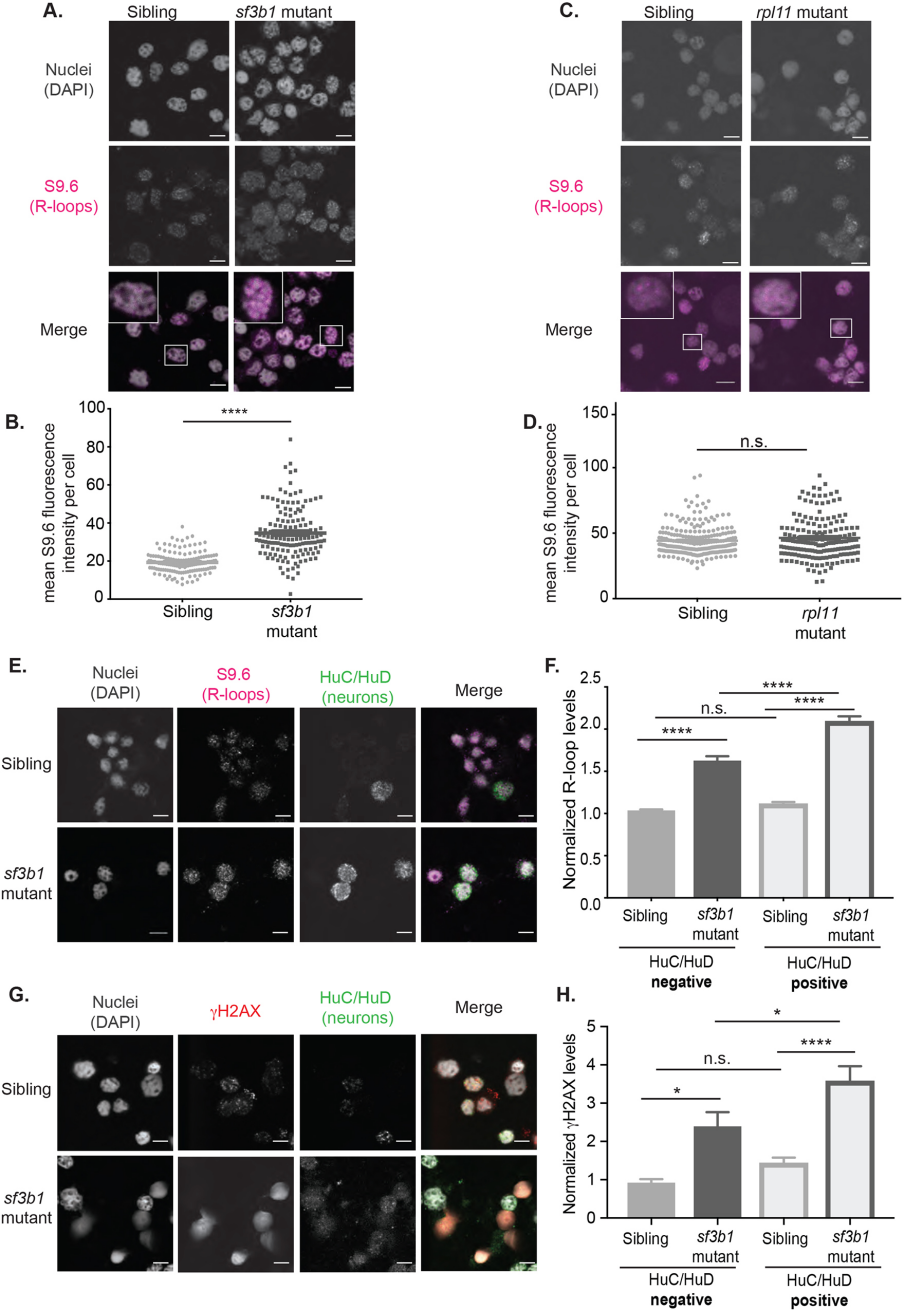
**R-loop levels are increased in spliceosomal mutant zebrafish.** (A) Immunofluorescence confocal images of nuclei (DAPI) and R-loops (S9.6) in cells isolated from 24 hpf *sf3b1^hi3394aTg^* mutants and their wild-type siblings. The lower panel is a merged image with an inset of a single cell to better show R-loop patterns. (B) Quantification of R-loop levels in A. Each dot represents R-loop levels for a single cell. *P*-value calculated by *t*-test with Welch's correction. (C,D) R-loop levels in *rpl11^hi3820bTg^* mutants were measured as in A and B. (E) Co-staining of R-loops with the pan-neuronal marker HuC/HuD in single cells isolated from 24 hpf *sf3b1^hi3394aTg^* mutants and their wild-type siblings. (F) Quantification of R-loop levels in HuC/HuD-positive and HuC/HuD-negative cells. (G) Co-staining of γH2AX and HuC/HuD. (H) Quantification of γH2AX fluorescence intensity in HuC/HuD-positive and HuC/HuD-negative cells. For all graphs, error bars represent the s.e.m. For images, 63× magnification with 6.5× zoom. Scale bar: 5 μm. For F and H, *P*-values were calculated by one-way ANOVA with Sidak's multi-testing correction. *P<0.05; **P<0.01; ***P<0.001; **** *P*<0.0001; n.s., not significant. Data represent normalized values across three replicates.

On the basis of studies performed in human cell lines (Paulsen et al., 2009), we hypothesized that elevated R-loop levels are driving the accumulation of DNA damage in the splicing factor mutants. R-loops were examined specifically in the *sf3b1^hi3394aTg^* mutants for two reasons: firstly, because Sf3b1 is a core component of the spliceosome and, secondly, because Ccdc94, Sfpq and Plrg1 also have splicing-independent cellular functions that could potentially complicate the analysis of R-loops in their respective mutant lines (Mahajan, 2016; An et al., 2014; Shav-Tal and Zipori, 2002; Berry and Gould, 1997; Legerski, 2009; Vieira et al., 2006). To measure cellular R-loop levels, immunofluorescence was performed using the S9.6 monoclonal antibody, which recognizes RNA:DNA hybrid structures (Boguslawski et al., 1986). Nuclear R-loop levels were quantified in single cells isolated from 24 hpf *sf3b1^hi3394aTg^* mutants and pools of wild-type and heterozygous siblings, which are phenotypically indistinguishable. Indeed, the mutants showed a significant increase in RNA:DNA hybrid levels compared with siblings (Fig. 3A,B). Importantly, R-loops were not simply a consequence of a loss of housekeeping genes, as *rpl11^hi3820bTg^* failed to show increased R-loop levels compared with sibling controls (Fig. 3C,D). As extensive apoptosis is observed in the neuronal tissue of splicing factor mutants, we asked whether R-loop accumulation is elevated in neuronal cells. To investigate this, we co-stained single cells isolated from 24 hpf *sf3b1^hi3394aTg^* mutants and wild-type siblings with the S9.6 RNA:DNA hybrid antibody and the neuronal-specific HuC/HuD antibody (Marusich et al., 1994). We next analyzed and compared R-loop levels between HuC/HuD-positive and HuC/HuD-negative cells, and observed a significant increase in R-loop levels in HuC/HuD-positive *sf3b1^hi3394aTg^* mutant cells compared with negative cells (Fig. 3E,F). Consistent with this observation, elevated levels of DNA DSBs were also found in embryonic neurons, as demonstrated by the concomitant enrichment of γH2AX in HuC/HuD-positive *sf3b1^hi3394aTg^* mutant cells compared with negative cells (Fig. 3G,H). Thus, based on S9.6-positive antibody staining, which is the established method for detecting R-loops, these data show that neurons are especially likely to accumulate R-loops in splicing factor mutants.

To independently confirm R-loop detection with the S9.6 antibody, we took advantage of recent findings that R-loop-driven DNA damage is associated with an increase in levels of histone H3 phosphorylated at serine 10 (H3S10P) (Castellano-Pozo et al., 2013; Garcia-Pichardo et al., 2017). On the basis of these studies, H3S10P levels were examined in *sf3b1^hi3394aTg^* mutants using whole-mount immunofluorescence (Fig. S3). Indeed, elevated levels of H3S10P were observed in neural tissues in *sf3b1^hi3394aTg^* mutants compared with siblings. Taken together, these data show that an early response to splicing factor depletion in zebrafish embryonic neurons is increased R-loop formation, which in turn promotes DNA damage and sensitivity to apoptotic stimuli.

### RNASEH1 reverses R-loop accumulation, DNA DSBs and neuronal apoptosis in *sf3b1* mutants

**Fig. 4.**
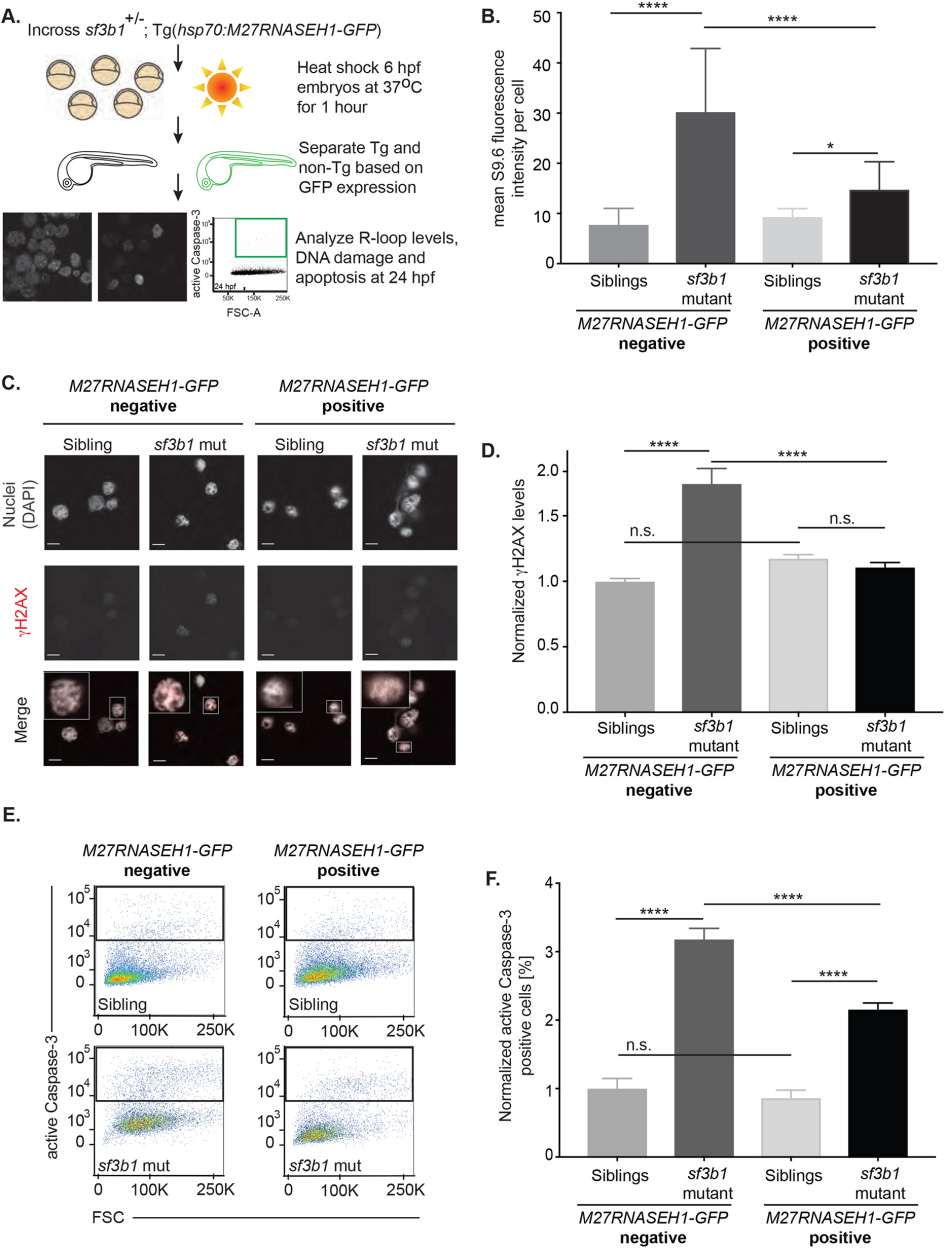
**RNASEH1 reverses R-loops, DNA DSBs and apoptosis in spliceosomal mutant zebrafish.** (A) Schematic of the RNASEH1 overexpression experiment. (B) Quantification of R-loop levels in cells isolated from 24 hpf *Tg(hsp70:M27RNASEH1-GFP)*-negative and *Tg(hsp70:M27RNASEH1-GFP)*-positive *sf3b1^hi3394aTg^* mutant and wild-type siblings following heat shock induction. (C) Confocal images showing immunofluorescence of nuclei (DAPI) and DNA damage (γH2AX) in cells isolated from 24 hpf *Tg(hsp70:M27RNASEH1-GFP)*-negative and *Tg(hsp70:M27RNASEH1-GFP)*-positive *sf3b1^hi3394aTg^* mutant and wild-type siblings. The lower panel is a merged image with an inset of a single cell to better show γH2AX staining. (D) Quantification of γH2AX fluorescence intensity in *sf3b1^hi3394aTg^* mutant and wild-type siblings. (E) Representative flow cytometry plots showing active caspase-3 levels on the *y*-axis and forward scatter (FSC) on the *x*-axis. (F) Quantification of active caspase-3 levels in 24 hpf *Tg(hsp70:M27RNASEH1-GFP)*-negative and *Tg(hsp70:M27RNASEH1-GFP)*-positive *sf3b1^hi3394aTg^* mutant and wild-type siblings following heat shock induction. For images, 63× magnification with 6.5× zoom. Scale bars: 5 μm. Error bars represent s.e.m. *P*-values were calculated by one-way ANOVA with Sidak's multi-testing correction. *P<0.05; **P<0.01; ***P<0.001; **** *P*<0.0001; n.s., not significant. For D and F, data represent normalized values across three replicates.

To resolve R-loop structures, cells utilize ribonuclease H enzymes that specifically cleave the RNA in an RNA:DNA hybrid (Santos-Pereira and Aguilera, 2015). To develop a system in which we could test whether unresolved R-loops functionally mediate the tissue-specific phenotypes associated with splicing factor mutants, we generated a transgenic zebrafish system for inducible expression of ribonuclease HI (RNASEH1)*.* We made an expression construct encoding human RNASEH1 lacking the first 26 amino acids that are required for mitochondrial localization (Fig. 4A) (Suzuki et al., 2010). This truncated version (M27RNASEH1) circumvents potential RNASEH1 toxicity without affecting its ability to resolve R-loops in the nucleus (Paulsen et al., 2009). A transgenic line was generated in which the truncated protein was expressed as a fusion with green fluorescent protein (GFP). Expression of this *M27RNASEH1-GFP* fusion gene can be induced by heat shock (Halloran et al., 2000) and subsequently visualized throughout the embryo, called *Tg(hsp70:M27RNASEH1-GFP)*. To determine whether expression of M27RNASEH1-GFP could diminish R-loop levels in *sf3b1^hi3394aTg^* mutants, we incrossed *sf3b1^hi3394aTg/+^*; *Tg(hsp70:M27RNASEH1-GFP)* animals, exposed the embryos to heat shock at 6 hpf, and then analyzed R-loop levels at 24 hpf (Fig. 4A,B). Using S9.6 fluorescence intensity to measure R-loops, it was found that forced expression of *M27RNASEH1-GFP* significantly reversed the accumulation of R-loops to nearly wild-type levels in *sf3b1^hi3394aTg^* mutants (Fig. 4B), indicating that M27RNASEH1-GFP can functionally resolve aberrant R-loop accumulation *in vivo*.

As RNASEH1 expression can suppress DNA damage caused by aberrant R-loop formation in cell culture experiments (Paulsen et al., 2009), we tested whether M27RNASEH1-GFP could reverse the accumulation of DNA DSBs and elevated apoptosis in *sf3b1^hi3394aTg^* mutants. We therefore incrossed *sf3b1^hi3394aTg/+^*; *Tg(hsp70:M27RNASEH1-GFP)* and analyzed γH2AX protein levels (by single-cell immunofluorescence) and apoptosis levels (by flow cytometry) in GFP-negative and GFP-positive progeny at 24 hpf (Fig. 4A,C-F). We found that expression of M27RNASEH1-GFP reversed the accumulation of γH2AX in *sf3b1^hi3394aTg^* mutant embryos to levels observed in wild-type and heterozygous siblings (Fig. 4C,D). Using a sensitive and quantitative flow cytometry assay to measure active caspase-3 levels (see Materials and Methods), we also observed a significant, albeit incomplete, suppression of apoptosis in *sf3b1^hi3394aTg^* mutants in response to M27RNASEH1-GFP expression, compared with GFP-negative siblings (Fig. 4E,F). However, rescue of apoptosis and DNA damage was not associated with reversal of the gross morphological defects observed in *sf3b1^hi3394aTg^* mutants. This result is similar to that observed for *tp53^zdf1^*; *sf3b1^hi3394aTg^* double mutants, which show suppressed apoptosis but an incomplete reversal of all morphological defects (Fig. 1B, Fig. S2A and data not shown). Thus, although our results suggest an immediate response to splicing factor dysfunction is accumulation of R-loops and DNA damage, later roles for these factors in mRNA processing and alternative splicing are likely to be crucial for morphogenesis events and embryonic survival. Nonetheless, these findings suggest that at least one mechanism underlying the neuronal tissue specificity associated with splicing factor mutations is sensitivity to R-loop accumulation and increased DNA damage.

## DISCUSSION

Formation of the embryonic nervous system requires rapid proliferation of neural progenitors that is coordinated with differentiation of distinct neural and glial cell types. To achieve this, molecular mechanisms must ensure genetic material is faithfully replicated to daughter cells and that gene expression is tightly regulated to produce the correct numbers of neurons and glia. Disrupting such mechanisms is expected to cause diminished neuronal cell survival and/or inappropriate differentiation that can lead to neurological diseases and/or neurodegeneration. Our previous studies in zebrafish identified members of the splicing machinery, Ccdc94 and Plrg1, to be crucial in regulating neuronal survival during embryogenesis (Sorrells et al., 2012). Although the *ccdc94* and *plrg1* mutants did not display significant defects in splicing of apoptotic factors, they did exhibit a significant elevation of *tp53* mRNA and protein levels. These findings supported a model in which splicing factor inhibition leads to increased *tp53* levels that in turn sensitizes neurons to apoptotic stimuli, such as IR. In this study, we tested this model using genetic mutants with elevated *tp53* mRNA and/or protein levels, including other core components of the splicing machinery such as *sf3b1*, and found that neuronal sensitivity to IR did not correlate with *tp53* levels. Instead, splicing factor mutants were found to show an increase in the phosphorylation of the DNA-damage-associated histone γH2AX that occurs independent of Tp53. We further show that an elevation in R-loop levels, at least in part, causes this increase in DNA damage in neuronal cells. Thus, a major finding of this study is that control of R-loop levels in neuronal cells during embryogenesis is crucial for formation of the nervous system. By contrast, other developing tissues appear to be more tolerant of R-loop dysfunction, a finding revealed through whole-animal approaches that helps to clarify why splicing factor mutations are associated with specific diseases in humans, including neurodegeneration. The zebrafish splicing factor mutants, coupled with the newly developed conditional transgenic line expressing RNASEH1, will allow researchers to further delineate the relative contribution of R-loop-dependent and R-loop-independent effects of the spliceosome on genomic integrity and tissue maintenance.

We previously proposed that embryonic neurons are selectively sensitive to IR because they proliferate more rapidly than other cell types at the developmental time points analyzed (Sorrells et al., 2012). The finding that R-loops preferentially accumulate in neurons further support this notion and suggest that increased transcriptional activity in embryonic neuronal cells might also have a role. In agreement with this hypothesis is the prevalent model that R-loop-mediated DNA DSBs are caused by collisions that occur between the RNA polymerase machinery during transcription and the DNA replication machinery during S phase (Hamperl et al., 2017). R-loop formation strongly correlates with high rates of transcription (Sanz et al., 2016) and neurons are highly transcriptionally active, often transcribing large genes (>200 kb) that are prone to R-loop accumulation (King et al., 2013; Sanz et al., 2016). Additionally, R-loop formation is modulated by histone modifications and chromatin state (Chédin, 2016), both of which are highly cell-type specific. Together with our *in vivo* data, these studies suggest that proliferation status, transcriptional rates and chromatin pattern are the driving forces behind the selectivity of embryonic neurons to DNA damage and apoptosis in splicing factor mutants.

Our studies on Tp53 demonstrated that elevated levels of Tp53 were neither necessary nor sufficient for mediating the increased DNA damage or radiosensitivity; however, Tp53 is required for executing the final apoptotic program. Indeed, our previous study showed that Tp53 acts upstream of apoptosis mediated by B-cell lymphoma 2 (Bcl2), as *bcl2* mRNA overexpression also rescues cell death in *ccdc94* mutants (Sorrells et al., 2012). Together these findings begin to delineate a genetic pathway that connects splicing-factor-induced R-loop formation to neuronal apoptosis through Tp53. However, these studies also indicate that another molecule(s) is required to sense and/or transmit the increased DNA damage in splicing mutants to Tp53 and Bcl2 to execute apoptosis. Two major DNA damage sensing kinases that could be explored in future studies include Ataxia-Telangiectasia Mutated (ATM) and Ataxia-Telangiectasia and Rad3-related (ATR), as these have been linked to R-loop-mediated DNA damage (Hamperl et al., 2017; Sollier and Cimprich, 2015; Tresini et al., 2015). For example, R-loop-driven DNA DSBs in proliferating cells often occur at sites of replication fork stalling, which are sensed by ATR (Hamperl et al., 2017; Sollier and Cimprich, 2015). Recent work has also demonstrated that spliceosome displacement from pre-mRNA results in R-loops that then activate ATM signaling at transcription-blocking lesions, leading to the induction of alternative splicing and transcriptional changes to respond to the DNA damage (Tresini et al., 2015). ATM is mutated in the neurodegenerative disease Ataxia-Telangiectasia, thus providing another potential link between R-loops and neuronal health. The zebrafish system is ideal for testing such hypotheses and further delineating the splicing-factor-induced neuronal apoptosis pathway through generation of CRISPR/Cas9 mutants in DNA damage sensing kinases combined with double/triple mutant analysis with the established splicing factor and/or *tp53* mutants.

Conditional expression of *M27RNASEH1* only partially suppressed neuronal apoptosis in the *sf3b1^hi3394aTg^* mutants, suggesting that either the levels of exogenous RNASEH1 were insufficient to resolve all R-loops or that R-loop-independent mechanisms are partly responsible for the mutant phenotypes. Most notably, it is possible that the well-established function of splicing factors in pre-mRNA processing could also drive apoptosis and developmental defects in splicing factor mutants. In our original analysis of *ccdc94^zd1000^* mutants, we noted few changes in splicing or gene expression at the developmental stages that were sensitive to IR. In particular, we carefully examined potential splice variants in anti- and pro-apoptotic factors (*bcl2*, *mcl1*, *bax*, etc.); however, we did not find any differences between mutant and wild-type embryos at 30 hpf. Similarly, our unpublished RNA sequencing of *sf3b1^hi3394aTg^* mutants at 20 hpf indicated no splicing alterations in apoptotic factors (data not shown). The lack of splicing defects is probably due to perdurance of maternal *ccdc94* and *sf3b1* mRNAs, as detected in the respective RNA sequencing experiments. However, even when apoptosis is suppressed in splicing factor mutants by exogenous RNASEH1 expression, *tp53* loss or *bcl2* mRNA overexpression, these embryos still display morphological phenotypes and embryonic lethality, which we believe is due to the canonical role of these factors in mRNA splicing. Thus, our findings indicate that R-loop formation is an immediate response to impaired splicing and/or occurs when splicing efficiency is limited but not completely abolished, which in turn induces neuronal apoptosis. The inability of *M27RNASEH1* overexpression to completely block apoptosis in these mutants is probably due to the role of splicing factors in mRNA processing and/or alternative splicing.

In addition to neurodegenerative diseases, spliceosomal components are commonly mutated in cancers, with the prognostic indication of the mutation being heavily influenced by tissue type (Dvinge et al., 2016). Our data suggest that cell-type-specific regulation of R-loops by splicing factors or disparate cellular responses to aberrant R-loops could contribute to the tissue-selective differences in cancers caused by splicing mutations. Moreover, for tissues that respond to splicing factor defects in a similar way to embryonic neurons, it is possible that unresolved R-loop-mediated DNA damage could amplify mutagenesis over time contributing to disease pathogenesis. Our data present the paradigm that pushing the system into overdrive through the increased induction of DNA damage or by further perturbing R-loop levels would be therapeutically beneficial to enhance tumor cell death when the spliceosome is altered. Importantly, drugs that target splicing factors are being developed for the treatment of cancer and our studies suggest that a therapeutic index could be readily established that allows increased DNA damage and apoptosis in highly proliferating cancer cells without affecting overt splicing in normal tissues.

## MATERIALS AND METHODS

### Zebrafish

All experiments were performed in strict accordance with the recommendations in the Guide for the Care and Use of Laboratory Animals of the NIH, and by the University of Utah and Albert Einstein College of Medicine Institutional Animal Care and Use Committee (IACUC). All efforts were made to minimize suffering. Zebrafish were maintained, bred and developmentally staged as described (Westerfield, 2000). Wild-type and *txnl4a^zd1006^* embryos were derived from the AB strain. The *plrg1^hi3174aTg^*, *sf3b1^hi3394aTg^*, *sfpq^hi1779Tg^*, *rpl11^hi3820bTg^*, *hspa8^hi138Tg^* and *terfa^hi3678Tg^* lines (Amsterdam et al., 2004) were obtained from the Zebrafish International Resource Center. All lines were outcrossed to the AB strain for at least seven generations. The *p53^zdf1/zdf1^* and *ccdc94^zd1000^* (*rs7*) lines were described previously (Berghmans et al., 2005; Sorrells et al., 2012). All experiments with the *p53^zdf1/zdf1^* line were performed with homozygous parents, thus removing any potential for maternal contribution of wild-type Tp53.

### Zebrafish line generation and identification

The *rs25* (*txnl4a^zd1006^*) line was identified in an F3 forward genetic screen as described (Sorrells et al., 2012). To identify the genetic mutation that caused the *rs25* phenotype, genetic linkage analysis was performed using microsatellite markers. The *rs25* mutation was found to reside on linkage group 19 between markers z26232 and z9059 (Fig. S1E). To identify candidate *rs25* mutations on chromosome 19, 4 dpf *rs25* siblings and mutants were compared by RNA sequencing. Analysis of chromosome 19 yielded 75 single-nucleotide polymorphisms that were present in *rs25* mutants but not in *rs25* siblings or wild-type sequence from the Sanger database.

The *Tg(hsp70:M27RNASEH1-GFP)* strain, which is designated *zd1009Tg* with the construct named *Tg(hsp70I:Has.RNASEH1_M1_G26del-GFP)* in ZFIN.org, was generated using human *RNASEH1* cDNA lacking the 5′ mitochondrial localization sequence, starting with Met27 and removing the stop codon to allow fusion to GFP. This construct was cloned into the middle-entry pDONR221 Gateway vector (Invitrogen). The zebrafish *hsp70* promoter in a 5′ Gateway entry vector and the eGFP polyA in a 3′ Gateway entry vector was obtained from the Kwan laboratory (Kwan et al., 2007). The *hsp70:M27RNASEH1-GFP* clone was created by Gateway LR reaction of entry vectors containing the zebrafish *hsp70* promoter, the human *M27RNASEH1* construct, *GFP* and *pDestTol2-crystallin-YFP* (http://tol2kit.genetics.utah.edu/index.php/Main_Page). Clones were verified by Sanger sequencing. Transposase mRNA (500 pl) and plasmid DNA (400 pl) were injected at one-cell stage and embryos were screened for yellow fluorescent protein (YFP)-positive eyes at 3 dpf. Adults were crossed and screened for germline transmission of the eye marker and whole-body GFP expression 1 h after heat shock at 37°C for 60 min.

### Genotyping

Embryos and adults were tail or fin clipped, respectively, to obtain genomic DNA for genotyping. *ccdc94^zd1000^* mutations were analyzed by high-resolution melt analysis. The *plrg1^hi3174aT^^g^*, *sf3b1^hi3394aTg^*, *sfpq^hi1779Tg^*, *rpl11^hi3820bTg^*, *hspa8^hi138Tg^*, *terfa^hi3678Tg^* and *p53^zdf1^* mutations were analyzed by PCR and gel electrophoresis.

### Zebrafish embryo microinjections

Zebrafish embryos were injected at the one-cell stage with 500 pl of mRNA or 20 µM mismatch morpholino or *plrg1* morpholino (Kleinridders et al., 2009). DNA vectors were cleaned using Denville spinsmart plasmid mini-prep columns (CM-400) and Qiagen buffers, following the QIAprep Spin Miniprep kit handbook (Qiagen). RNA was generated by linearizing *pCS2+Transposase* vector with *Not1* and *in vitro* transcription with SP6 mMESSAGE Machine kit (Ambion, AM1340). RNA was cleaned by NucAway Spin Columns (Ambion, AM10070).

### Irradiator usage

IR was administered with either a Cs-137 γ-irradiator (Gammacell 1000) for Fig. S1A-C or an X-ray irradiator (RadSource RS2000) for all other figures.

### Whole-mount *in situ* hybridization

*In situ* RNA probe vectors were generated as described previously (Sorrells et al., 2012). Full-length antisense *p53* and *puma* probes were generated by digesting with *Apa*1 or *Nco*1, respectively, and cleaned with PCR purification columns (Denville CM500-250) using Qiagen buffers, following the QIAquick PCR purification kit protocol (Qiagen 28104). Clean, linearized DNA was transcribed with SP6 for antisense probes (Ambion AM2071) and T7 for sense probes (Ambion AM2082) with digoxygenin-RNA-labeling mix (Roche 11277073910). Unincorporated nucleotides were removed using Ambion NucAway Spin Columns (AM10070).

Embryos were dechorionated, staged and fixed overnight in 4% paraformaldehyde at 4°C, with rocking. Fixed embryos were washed three times with 1× phosphate-buffered saline (PBS) plus 0.1% Tween 20 (PBST) at room temperature for 5 min each and then dehydrated in 100% methanol at −20°C for a minimum of 2 h. Embryos were rehydrated by washing three times in PBST for 5 min each at room temperature and incubated in hyb-minus [50% formamide, 5× saline sodium citrate (SSC) and 0.1% Tween 20] for 15-60 min at 68°C, with rocking. Embryos were then incubated in hyb-plus (hyb-minus, 5 mg ml^−1^ torula RNA type VI, 50 μg ml^−1^ heparin) for 3 h at 68°C, with rocking. RNA probe at a concentration of 1 ng μl^−1^ was added to the embryos in hyb-plus and incubated overnight at 68°C, with rocking. Embryos were then washed at 68°C, with rocking, using a series of washes: twice with 2× SSCT-formamide (2× SSC, 0.1% Tween 20, 50% formamide) for 30 min, once with 2× SSCT for 15 min and then twice with 0.2× SSCT for 30 min. Embryos were then washed three times for 5 min each at room temperature in MABT (100 mM maleic acid, 150 mM sodium chloride, 100 mM Tris pH 9.5, 0.1% Tween 20) and blocked for 1 h [MABT, 2% blocking reagent (Roche 11096176001) and 1% fetal bovine serum] at room temperature. Embryos were incubated overnight in anti-digoxigenin alkaline phosphatase FAB fragments (Roche 11093274910) at 1:5000 in block, rocking at 4°C. Embryos were washed twice in MABT for 1 h at room temperature and then washed in 0.1 M Tris pH 9.5 three times for 5 min at room temperature. Embryos were stained with Vector BCIP/NBT alkaline phosphatase (Vector SK5400) in 0.1 M Tris pH 9.5. To end staining, embryos were washed three times for 5 min each in PBST. Embryos were stored in PBST at 4°C until they were imaged in 4% methylcellulose and subsequently genotyped.

### Image acquisition and processing

Brightfield images were acquired using a Nikon SMZ1500 microscope and Olympus DP72 camera. Fluorescent images were acquired on an Olympus SZX16 microscope with a QImaging EXi Aqua black and white camera. Confocal images for zebrafish single-cell immunofluorescence were acquired using a Leica SP5 AOBS Inverted DMI6000 microscope with a 63× oil objective and zoomed in fields were taken at 6.5× zoom. FIJI, ImageJ, Flowjo, GraphPad Prism 6, Adobe Photoshop CS5.1 and Adobe Illustrator CS5.1 were used to generate figures.

### Whole-mount immunofluorescence and quantitation

Whole-mount active caspase-3 immunofluorescence was performed and quantified as described previously (Sorrells et al., 2012, 2013) with minor modifications, detailed below. Quantitation was performed after immunofluorescence by imaging tails in 4% methylcellulose on a depression slide. Representative images from at least three experiments are shown. To genotype animals, embryos were removed from methylcellulose with a metal probe and transferred to 15 µl alkaline lysis buffer (25 mM sodium hydroxide, 0.2 mM disodium EDTA) and incubated at 95°C for 2 h before adding 15 µl neutralizing buffer (40 mM Tris-hydrochloride). Genotyping was performed per specific genotype protocol. Caspase-3 quantitation represents the measurement of fluorescence intensity. Changes in fluorescence intensity probably represent both an increase in the number of apoptotic cells, as well as an increase in caspase-3 activation within individual cells. Whole-mount H3S10P immunofluorescence was performed using the same protocol as for activated caspase-3 immunofluorescence, but using an antibody that detected histone H3 phosphorylation on Ser10 (Santa Cruz SC8656). In experiments with extreme outliers, the embryos with the highest and lowest fluorescence intensity were removed from the analysis in each condition and each genotype. Statistical analysis was performed on at least ten embryos per condition. Image contrast was often altered for reader clarity and all composite images from the experiment were changed equally and simultaneously.

### Single-cell immunofluorescence of zebrafish embryonic cells

At 24 hpf, embryos were binned based on genotypic differences in morphology. To prepare single-cell suspensions, embryos were first dissociated with a razor blade while immersed in Dulbecco's phosphate-buffered saline (D-PBS), and then incubated with a 1:65 dilution of Liberase (Roche) at 37°C for 7 min. A 5% final concentration of fetal bovine serum was added to stop the enzymatic reaction. The single suspension was filtered through a 45 μM cell strainer and spun down at 900 ***g***. Cells were resuspended, counted and spotted onto a poly-L-lysine-coated slide and air-dried for ∼1 h. For S9.6, γH2AX and HuC/HuD staining, cells were fixed for 10 min with 4% paraformaldehyde (PFA) and permeabilized with ice-cold methanol for 5 min at −20°C. Cells were washed in 0.1% Tween 20/PBS and blocked for 1 h at room temperature with 5% bovine serum albumin (BSA)/0.2% milk/PBS. The mouse monoclonal S9.6 antibody was generated from the HB-8730 hybridoma (Boguslawski et al., 1986) by the Einstein Hybridoma Facility and purified by the Macromolecular Therapeutics Facility (Nadel et al., 2015). The S9.6 primary antibody was diluted 1:50, the rabbit polyclonal anti-zebrafish γH2AX primary antibody (GeneTex GTX127342) was diluted 1:500 and the HuC/HuD mouse monoclonal antibody (Invitrogen A21271) was diluted 1:100. All were diluted in blocking buffer and incubated on cells for 3 h at room temperature. Secondary antibodies (goat anti-mouse AlexaFluor-488, goat anti-mouse AlexaFluor-488 IgG2a, goat anti-mouse AlexaFluor-488 IgG2b or goat anti-rabbit AlexaFluor-594, Thermo Fisher Scientific) were diluted 1:1000 and incubated for 1 h at room temperature. After final washes, cells were mounted with DAPI-Fluoromount-G (Southern Biotech), covered with a 25×25 mm glass coverslip and sealed with clear nail polish around the corners. Fluorescence intensity measurements of S9.6 staining and counting of γH2AX-positive cells were performed using FIJI.

### Active caspase-3 intracellular flow-cytometry staining

At 24 hpf, embryos were binned based on genotypic differences in morphology and processed into single-cell suspensions, as described above. After the first spin at 900 ***g***, the pellet was resuspended in 100 µl 4% PFA and fixed for 15 min at room temperature. Cells were washed with 1× D-PBS and permeabilized in a saponin-based wash buffer (0.1% saponin/1% BSA/D-PBS) for 15 min at room temperature. Cells were incubated with active caspase-3 antibody (BD Biosciences) at a 1:500 dilution in saponin-based wash buffer for 30 min at room temperature. Secondary antibody incubations were performed at 1:1000 (goat anti-rabbit AlexaFluor-594) for 30 min in the dark. Cells were resuspended in wash buffer and analyzed by flow cytometry using a BD LSRII-Yellow Analyzer. Quantitations were made using FlowJo 10.3 beta software.

### Quantitative real-time PCR

Embryos were pooled for RNA collection on the basis of genotypic differences in morphology. If there was no obvious mutant phenotype, tail clips were taken to genotype the embryos. The remaining animal body was placed in RLT buffer, stored at −80°C and pooled after genotyping. RNA was isolated from a minimum of 10-50 embryos per sample using the Qiagen RNeasy kit (74104). On-column DNase treatment was performed using the Qiagen RNase-Free DNase set (79254). Purified RNA (500 ng to 1 µg) was used to generate cDNA using the Roche Transcriptor First Strand cDNA Synthesis Kit (04379012001). For mRNA analysis, cDNA was generated using oligo-dT primers. For pre-mRNA analysis, cDNA was generated using random hexamer primers. The cDNA was diluted 1:10 in nuclease-free water, and three technical replicates were analyzed using the Roche LightCycler 480 Probes Master PCR mix (04887301001) on an Eppendorf Realplex Mastercycler. Primers were designed by Roche to be used with their Universal Probe Library. Primer design was previously described (Sorrells et al., 2012). Data were averaged from three independent biological experiments.

### Western analysis

Embryos were pooled for protein collection on the basis of phenotype. If there was no obvious phenotype, tail clips were taken to genotype the embryos. The remaining animal body was placed in modified RIPA buffer (1× PBS, 1% Nonide P-40, 0.5% sodium deoxycholate, 0.2% sodium dodecyl sulfate) with additives [1% protease inhibitors (Sigma P8340), 10 mM sodium fluoride, 1 mM sodium orthovanadate and 5 mM EDTA] and stored at −80°C until completion of genotyping. Genotypes were pooled and samples homogenized by mortar and pestle (Thermo Fisher Scientific K7495211590) on ice. Protein concentration was determined by BCA Protein Assay (Pierce 23227). A total of 25 ng protein was added to 4× NuPAGE LDS sample buffer (Life Technologies 1606369) with 40 mM DTT in RIPA and heated to 99°C for 10 min. Denatured protein was loaded onto a denaturing gel (Novex NP0301box) and transferred to a methanol-hydrated PVDF membrane (Immobilon-P IPVH00010). The membrane was blocked in 3% BSA (Amresco 0332) diluted in Tris-buffered saline plus 0.1% Tween 20 (TBST) and then probed with anti-GAPDH antibody (Abcam 9484) and anti-γH2AX (Cell Signaling Technology 2577 s). The blot was cut at approximately 25 kDa to facilitate multiple blots. The upper blot was incubated with anti-GAPDH at 1:2000 in 3% BSA and the lower blot was incubated with anti-γH2AX antibody at 1:2000 in 3% BSA, both with overnight incubation at 4°C. The blots were washed four times for 5 min each in Tris-buffered saline + 0.1% Tween 20 (TBST). Anti-mouse horseradish peroxidase secondary antibody (Cell Signaling Technology 7076S) was used at 1:5000 in 3% BSA for the anti-GAPDH antibody, rocking for 30 min at room temperature. Anti-rabbit horseradish peroxidase secondary antibody (Cell Signaling Technology 7074) was used at 1:5000 in 3% BSA for the anti-γH2AX antibody, rocking for 30 min at room temperature. The blots were washed four times for 10 min each in TBST before the addition of chemiluminescent horseradish peroxidase substrate (Millipore ABKLS0500) and detection of signal by film (Thermo Fisher Scientific 34090). If Tp53 was analyzed, the blot was stripped for 1 h rotating at 60°C (32.5 mM Tris, 2% sodium dodecyl sulfate, 100 mM 2-mercaptoethanol) in 50 ml conical tubes (Denville C1060-P). The blots were washed four times briefly with TBST in the conical tubes and transferred to a flat dish where they were washed four additional times in TBST for 5 min each. The blots were re-blocked in 10% non-fat dry milk diluted in TBST and probed using anti-Tp53 antibody. The zebrafish Tp53 antibody was kindly provided by Dr Lane at A*STAR, Singapore (Lee et al., 2008) and used at 1:100 in 10% milk with overnight incubation at 4°C. The anti-mouse secondary antibody and detection was performed as described above. Developed films were scanned at 600 dots per inch for figures.

### Statistics

Experiments were performed a minimum of three times with similar results. Statistical analyses were performed as indicated in each figure; error bars indicate the standard error of mean unless otherwise indicated.

## Supplementary Material

Supplementary information

## References

[DMM031583C1] Amsterdam A., Nissen R. M., Sun Z., Swindell E. C., Farrington S. and Hopkins N. (2004). Identification of 315 genes essential for early zebrafish development. *Proc. Natl. Acad. Sci. USA* 101, 12792-12797. 10.1073/pnas.0403929101 15256591 PMC516474

[DMM031583C2] An D., Kim K. and Lu W. (2014). Defective entry into mitosis 1 (Dim1) negatively regulates osteoclastogenesis by inhibiting the expression of nuclear factor of activated T-cells, cytoplasmic, calcineurin-dependent 1 (NFATc1). *J. Biol. Chem.* 289, 24366-24373. 10.1074/jbc.M114.563817 25023277 PMC4148864

[DMM031583C3] Berghmans S., Murphey R. D., Wienholds E., Neuberg D., Kutok J. L., Fletcher C. D. M., Morris J. P., Liu T. X., Schulte-Merker S., Kanki J. P. et al. (2005). tp53 mutant zebrafish develop malignant peripheral nerve sheath tumors. *Proc. Natl. Acad. Sci. USA* 102, 407-412. 10.1073/pnas.0406252102 15630097 PMC544293

[DMM031583C4] Berry L. D. and Gould K. L. (1997). Fission yeast dim1^+^ encodes a functionally conserved polypeptide essential for mitosis. *J. Cell Biol.* 137, 1337-1354. 10.1083/jcb.137.6.1337 9182666 PMC2132542

[DMM031583C5] Boguslawski S. J., Smith D. E., Michalak M. A., Mickelson K. E., Yehle C. O., Patterson W. L. and Carrico R. J. (1986). Characterization of monoclonal antibody to DNA.RNA and its application to immunodetection of hybrids. *J. Immunol. Methods* 89, 123-130. 10.1016/0022-1759(86)90040-2 2422282

[DMM031583C6] Castellano-Pozo M., Santos-Pereira J. M., Rondón A. G., Barroso S., Andújar E., Pérez-Alegre M., Garcia-Muse T. and Aguilera A. (2013). R loops are linked to histone H3 S10 phosphorylation and chromatin condensation. *Mol. Cell* 52, 583-590. 10.1016/j.molcel.2013.10.006 24211264

[DMM031583C7] Chédin F. (2016). Nascent connections: R-loops and chromatin patterning. *Trends Genet.* 32, 828-838. 10.1016/j.tig.2016.10.002 27793359 PMC5123964

[DMM031583C8] Dai C. and Gu W. (2010). p53 post-translational modification: deregulated in tumorigenesis. *Trends Mol. Med.* 16, 528-536. 10.1016/j.molmed.2010.09.002 20932800 PMC2978905

[DMM031583C9] Danilova N., Kumagai A. and Lin J. (2010). p53 upregulation is a frequent response to deficiency of cell-essential genes. *PLoS ONE* 5, e15938 10.1371/journal.pone.0015938 21209837 PMC3013139

[DMM031583C10] Danilova N., Sakamoto K. M. and Lin S. (2011). Ribosomal protein L11 mutation in zebrafish leads to haematopoietic and metabolic defects. *Br. J. Haematol.* 152, 217-228. 10.1111/j.1365-2141.2010.08396.x 21114664 PMC3457809

[DMM031583C11] De La Garza A., Cameron R. C., Nik S., Payne S. G. and Bowman T. V. (2016). Spliceosomal component Sf3b1 is essential for hematopoietic differentiation in zebrafish. *Exp. Hematol.* 44, 826-837.e4. 10.1016/j.exphem.2016.05.012 27260753 PMC4992596

[DMM031583C12] Dvinge H., Kim E., Abdel-Wahab O. and Bradley R. K. (2016). RNA splicing factors as oncoproteins and tumour suppressors. *Nat. Rev. Cancer* 16, 413-430. 10.1038/nrc.2016.51 27282250 PMC5094465

[DMM031583C13] Garcia-Pichardo D., Canas J. C., Garcia-Rubio M. L., Gomez-Gonzalez B., Rondon A. G. and Aguilera A. (2017). Histone mutants separate R loop formation from genome instability induction. *Mol. Cell* 66, 597-609.e5. 10.1016/j.molcel.2017.05.014 28575656

[DMM031583C14] Gottschalk A., Neubauer G., Banroques J., Mann M., Lührmann R. and Fabrizio P. (1999). Identification by mass spectrometry and functional analysis of novel proteins of the yeast [U4/U6.U5] tri-snRNP. *EMBO J.* 18, 4535-4548. 10.1093/emboj/18.16.4535 10449419 PMC1171528

[DMM031583C15] Groh M. and Gromak N. (2014). Out of balance: R-loops in human disease. *PLoS Genet.* 10, e1004630 10.1371/journal.pgen.1004630 25233079 PMC4169248

[DMM031583C16] Halloran M. C., Sato-Maeda M., Warren J. T., Su F., Lele Z., Krone P. H., Kuwada J. Y. and Shoji W. (2000). Laser-induced gene expression in specific cells of transgenic zebrafish. *Development* 127, 1953-1960. 10751183 10.1242/dev.127.9.1953

[DMM031583C17] Hamperl S., Bocek M. J., Saldivar J. C., Swigut T. and Cimprich K. A. (2017). Transcription-replication conflict orientation modulates R-loop levels and activates distinct DNA damage responses. *Cell* 170, 774-786.e19. 10.1016/j.cell.2017.07.043 28802045 PMC5570545

[DMM031583C18] Jette C. A., Flanagan A. M., Ryan J., Pyati U. J., Carbonneau S., Stewart R. A., Langenau D. M., Look A. T. and Letai A. (2008). BIM and other BCL-2 family proteins exhibit cross-species conservation of function between zebrafish and mammals. *Cell Death Differ.* 15, 1063-1072. 10.1038/cdd.2008.42 18404156 PMC3212414

[DMM031583C19] King I. F., Yandava C. N., Mabb A. M., Hsiao J. S., Huang H.-S., Pearson B. L., Calabrese J. M., Starmer J., Parker J. S., Magnuson T. et al. (2013). Topoisomerases facilitate transcription of long genes linked to autism. *Nature* 501, 58-62. 10.1038/nature12504 23995680 PMC3767287

[DMM031583C20] Kleinridders A., Pogoda H.-M., Irlenbusch S., Smyth N., Koncz C., Hammerschmidt M. and Bruning J. C. (2009). PLRG1 is an essential regulator of cell proliferation and apoptosis during vertebrate development and tissue homeostasis. *Mol. Cell. Biol.* 29, 3173-3185. 10.1128/MCB.01807-08 19307306 PMC2682009

[DMM031583C21] Kwan K. M., Fujimoto E., Grabher C., Mangum B. D., Hardy M. E., Campbell D. S., Parant J. M., Yost H. J., Kanki J. P. and Chien C.-B. (2007). The Tol2kit: a multisite gateway-based construction kit for Tol2 transposon transgenesis constructs. *Dev. Dyn.* 236, 3088-3099. 10.1002/dvdy.21343 17937395

[DMM031583C22] Lee K.-C., Goh W. L., Xu M., Kua N., Lunny D., Wong J. S., Coomber D., Vojtesek B., Lane E. B. and Lane D. P. (2008). Detection of the p53 response in zebrafish embryos using new monoclonal antibodies. *Oncogene* 27, 629-640. 10.1038/sj.onc.1210695 17684488

[DMM031583C23] Legerski R. J. (2009). The Pso4 complex splices into the DNA damage response. *Cell Cycle* 8, 3448-3449. 10.4161/cc.8.21.9760 19838058

[DMM031583C24] Mahajan K. (2016). hPso4/hPrp19: a critical component of DNA repair and DNA damage checkpoint complexes. *Oncogene* 35, 2279-2286. 10.1038/onc.2015.321 26364595

[DMM031583C25] Marusich M. F., Furneaux H. M., Henion P. D. and Weston J. A. (1994). Hu neuronal proteins are expressed in proliferating neurogenic cells. *J. Neurobiol.* 25, 143-155. 10.1002/neu.480250206 7517436

[DMM031583C26] Nadel J., Athanasiadou R., Lemetre C., Wijetunga N. A., Ó Broin P., Sato H., Zhang Z., Jeddeloh J., Montagna C., Golden A. et al. (2015). RNA:DNA hybrids in the human genome have distinctive nucleotide characteristics, chromatin composition, and transcriptional relationships. *Epigenetics Chromatin* 8, 46 10.1186/s13072-015-0040-6 26579211 PMC4647656

[DMM031583C27] Paulsen R. D., Soni D. V., Wollman R., Hahn A. T., Yee M.-C., Guan A., Hesley J. A., Miller S. C., Cromwell E. F., Solow-Cordero D. E. et al. (2009). A genome-wide siRNA screen reveals diverse cellular processes and pathways that mediate genome stability. *Mol. Cell* 35, 228-239. 10.1016/j.molcel.2009.06.021 19647519 PMC2772893

[DMM031583C28] Rogakou E. P., Pilch D. R., Orr A. H., Ivanova V. S. and Bonner W. M. (1998). DNA double-stranded breaks induce histone H2AX phosphorylation on serine 139. *J. Biol. Chem.* 273, 5858-5868. 10.1074/jbc.273.10.5858 9488723

[DMM031583C29] Santos-Pereira J. M. and Aguilera A. (2015). R loops: new modulators of genome dynamics and function. *Nat. Rev. Genet.* 16, 583-597. 10.1038/nrg3961 26370899

[DMM031583C30] Sanz L. A., Hartono S. R., Lim Y. W., Steyaert S., Rajpurkar A., Ginno P. A., Xu X. and Chédin F. (2016). Prevalent, dynamic, and conserved R-loop structures associate with specific epigenomic signatures in mammals. *Mol. Cell* 63, 167-178. 10.1016/j.molcel.2016.05.032 27373332 PMC4955522

[DMM031583C31] Shav-Tal Y. and Zipori D. (2002). PSF and p54^nrb^/NonO – multi-functional nuclear proteins. *FEBS Lett.* 531, 109-114. 10.1016/S0014-5793(02)03447-6 12417296

[DMM031583C32] Sollier J. and Cimprich K. A. (2015). Breaking bad: R-loops and genome integrity. *Trends Cell Biol.* 25, 514-522. 10.1016/j.tcb.2015.05.003 26045257 PMC4554970

[DMM031583C33] Sorrells S., Carbonneau S., Harrington E., Chen A. T., Hast B., Milash B., Pyati U., Major M. B., Zhou Y., Zon L. I. et al. (2012). Ccdc94 protects cells from ionizing radiation by inhibiting the expression of p53. *PLoS Genet.* 8, e1002922 10.1371/journal.pgen.1002922 22952453 PMC3431329

[DMM031583C34] Sorrells S., Toruno C., Stewart R. A. and Jette C. (2013). Analysis of apoptosis in zebrafish embryos by whole-mount immunofluorescence to detect activated Caspase 3. *J. Vis. Exp.* 82, e51060 10.3791/51060 PMC4109746 24378359

[DMM031583C35] Suzuki Y., Holmes J. B., Cerritelli S. M., Sakhuja K., Minczuk M., Holt I. J. and Crouch R. J. (2010). An upstream open reading frame and the context of the two AUG codons affect the abundance of mitochondrial and nuclear RNase H1. *Mol. Cell. Biol.* 30, 5123-5134. 10.1128/MCB.00619-10 20823270 PMC2953059

[DMM031583C36] Szafranski K., Abraham K. J. and Mekhail K. (2015). Non-coding RNA in neural function, disease, and aging. *Front. Genet.* 6, 87 10.3389/fgene.2015.00087 25806046 PMC4353379

[DMM031583C37] Tresini M., Warmerdam D. O., Kolovos P., Snijder L., Vrouwe M. G., Demmers J. A. A., Van Ijcken W. F. J., Grosveld F. G., Medema R. H., Hoeijmakers J. H. et al. (2015). The core spliceosome as target and effector of non-canonical ATM signalling. *Nature* 523, 53-58. 10.1038/nature14512 26106861 PMC4501432

[DMM031583C38] Vieira L., Sousa A. C., Matos P., Marques B., Alaiz H., Ribeiro M. J., Braga P., da Silva M. G. and Jordan P. (2006). Three-way translocation involves MLL, MLLT3, and a novel cell cycle control gene, FLJ10374, in the pathogenesis of acute myeloid leukemia with t(9;11;19)(p22;q23;p13.3). *CancerGenes, Chromosomes & Cancer* 45, 455-469. 10.1002/gcc.20311 16450356

[DMM031583C39] Westerfield M. (2000). *The Zebrafish Book - A Guide for the Laboratory Use of Zebrafish (Danio rerio)*. Eugene, Oregon: University of Oregon Press.

[DMM031583C40] Will C. L. and Lührmann R. (2011). Spliceosome structure and function. *Cold Spring Harb. Perspect. Biol.* 3, a003707 10.1101/cshperspect.a003707 21441581 PMC3119917

