# Supplementary material for: Spliceosomal components protect embryonic neurons from R-loop-mediated DNA damage and apoptosis
Source: Dis Model Mech. 2018 Feb 1;11(2):dmm031583. doi: 10.1242/dmm.031583 (PMC5894942; doi:10.1242/dmm.031583)
Supplement: Supplementary information [file dmm-11-031583-s1.pdf]

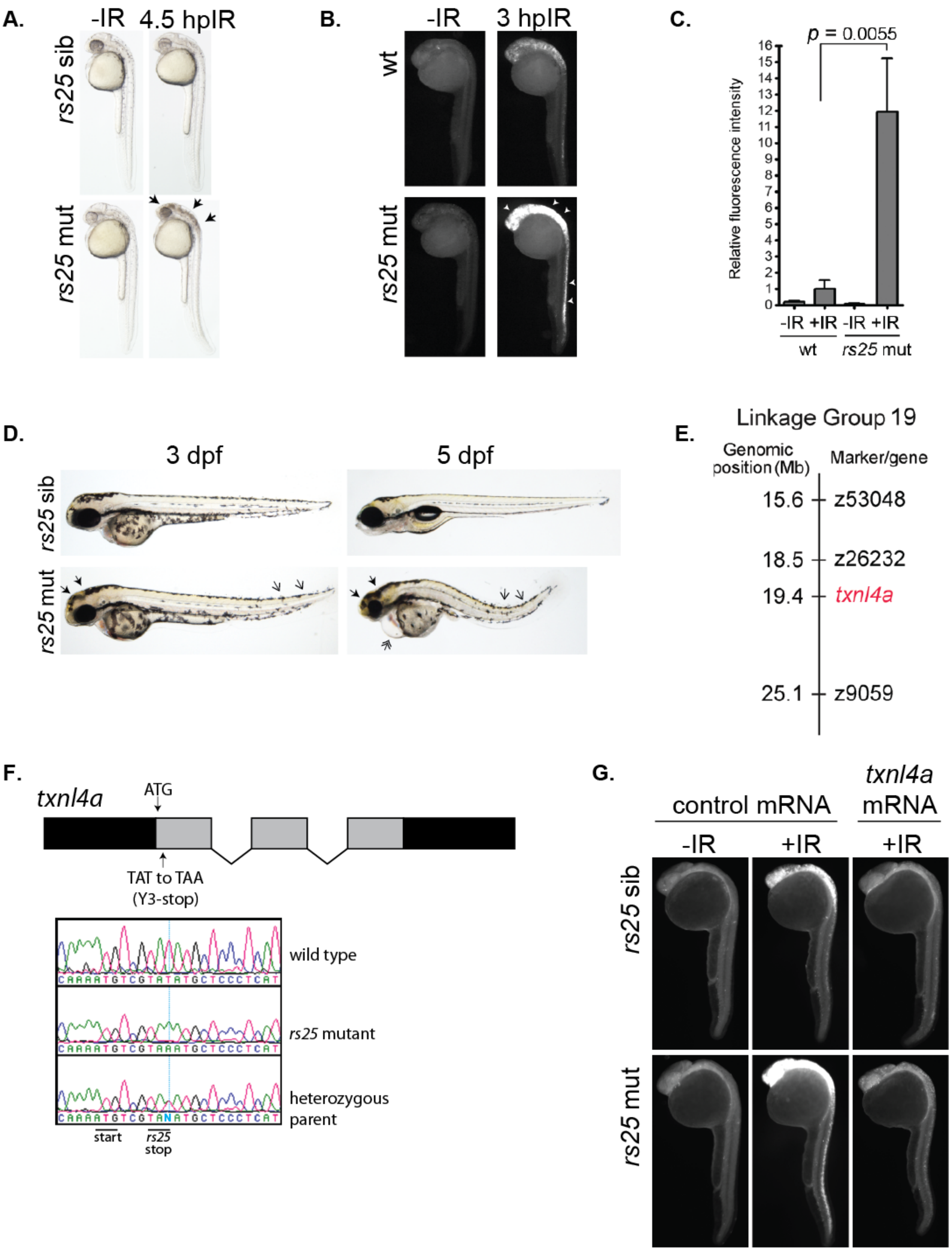

A.
-IR
4.5 hpIR
rs25 sib
rs25 mut
B.
-IR
3 hpIR
wt
rs25 mut
C.
p = 0.0055
Relative fluorescence intensity
-IR +IR -IR +IR
wt
rs25 mut
D.
3 dpf
5 dpf
rs25 sib
rs25 mut
E.
Linkage Group 19
Genomic position (Mb)
Marker/gene
15.6 z53048
18.5 z26232
19.4 txnl4a
25.1 z9059
F.
txnl4a
ATG
TAT to TAA (Y3-stop)
wild type
rs25 mutant
heterozygous parent
start
rs25 stop
G.
control mRNA
txnl4a mRNA
-IR
+IR
+IR
rs25 sib
rs25 mut

Figure S1

Figure S1. The *rs25* mutation disrupts the *txnl4a* gene

(A) *rs25* siblings and mutants were exposed to 10 Gy IR and analyzed by bright field microscopy at 4.5 hpIR. Black arrows in the *rs25* mutants mark the accumulation of cell death. **(B-C)** Wild type embryos and *rs25* mutants were exposed to 10 Gy IR at 24 hpf, fixed at 3 hpIR, analyzed by active Caspase-3 and fluorescence was quantified. White arrowheads in the *rs25* mutants mark the accumulation of active Caspase-3-marked apoptosis. **(D)** *rs25* siblings and mutants were imaged by bright field microscopy at 3 and 5 dpf. Solid arrowheads in *rs25* mutants mark accumulation of cell death in the head. Single, open arrowheads mark the curved tail, and double arrowheads mark heart edema. **(E)** The *rs25* mutation was localized to linkage group 19 between flanking markers z26232 and z9059. **(F)** Sequencing of *rs25* mutants revealed a thymine to adenine transition within the *txnl4a* coding sequence replacing the third amino acid (tyrosine) with a stop codon (Y3-Stop). Schematic of *txnl4a* shows 3 exons (in grey boxes), the 5' and 3' untranslated region (in black boxes), and introns (bent lines). Sequencing of the *txnl4a* gene is shown for wild type, *rs25* mutant, and an *rs25* heterozygote. A vertical blue line intersects the position of the mutated nucleotide. The solid, black lines under the sequencing designate the start of the coding sequence and *rs25*-mediated premature stop codon. **(G)** Embryos from an incross of *rs25* heterozygotes were injected with control mRNA (GFP) or *txnl4a* mRNA. Embryos were exposed (or left unexposed) to 10 Gy at 24 hpf and analyzed 3 hours later for active Caspase-3.

Disease Models & Mechanisms • Supplementary information

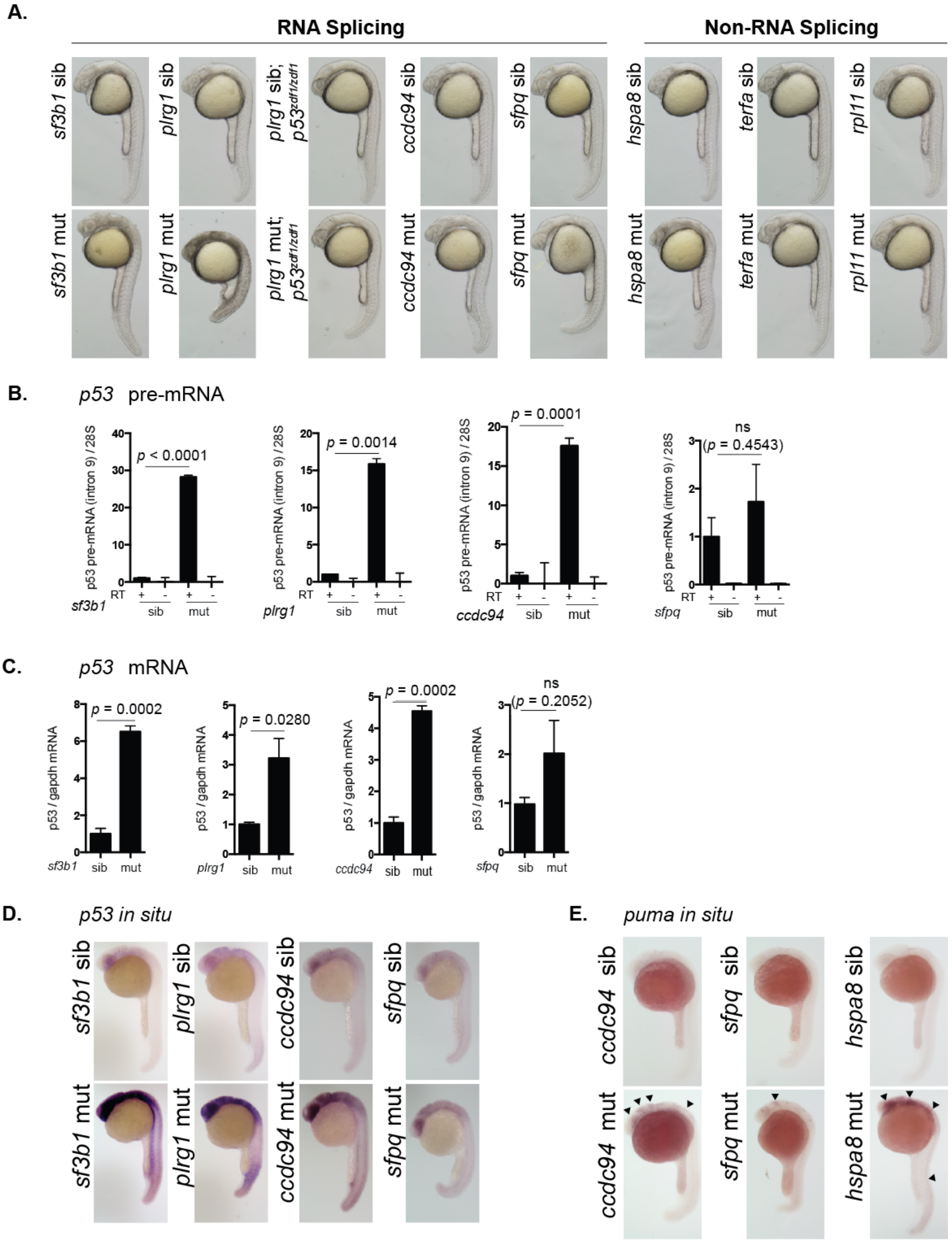

Figure S2

Disease Models & Mechanisms • Supplementary information

Figure S2. Splicing-factor mutants have elevated levels of *tp53*.

(A) Sibling and mutant embryos from *sf3b1*^{hi3394aTg}, *plrg1*^{hi3174aTg}, *plrg1*^{hi3174aTg};*p53*$^{zdf1/zdf1}$, *ccdc94*^{zd1000}, *sfpq*^{hi1779Tg}, *hspa8*^{hi138Tg}, *terfa*^{hi3678Tg}, and *rpl11*^{hi3820bTg} were imaged by bright-field microscopy at 25 hpf. Mutant phenotypes include neurodegeneration, curved tail, heart edema, and yolk extension defects. *terfa*^{hi3678Tg} and *rpl11*^{hi3820bTg} mutants are indistinguishable from siblings at this time point. **(B)** Splicing factor mutants and siblings were separated by phenotype. RNA was harvested from each group at 30 hpf, DNAse treated, and reverse transcribed using random hexamer primers. Intron 9 of *tp53* was analyzed by qPCR to determine levels of *tp53* pre-mRNA. Minus reverse transcriptase (-RT) samples were included to control for genomic DNA contamination. *tp53* pre-mRNA levels were normalized to *28S* RNA levels to yield a relative amount of RNA expression between groups. **(C)** RNA collected from embryos described in (B) was reverse transcribed using oligo-dT primers and analyzed by qPCR for *tp53* mRNA expression. *gapdh* mRNA levels were analyzed to determine relative amount of RNA expression per group. ns; not significantly different. **(D)** Splicing factor mutants and siblings were grown to 24 hpf and analyzed by whole mount *in situ* hybridization for *tp53* mRNA expression. **(E)** Sibling and mutant embryos from *ccdc94*^{zd1000}, *sfpq*^{hi1779Tg}, and *hspa8*^{hi138Tg} were grown to 24 hpf and analyzed by whole mount *in situ* hybridization for the mRNA expression of the Tp53-target gene *puma*.

Disease Models & Mechanisms • Supplementary information

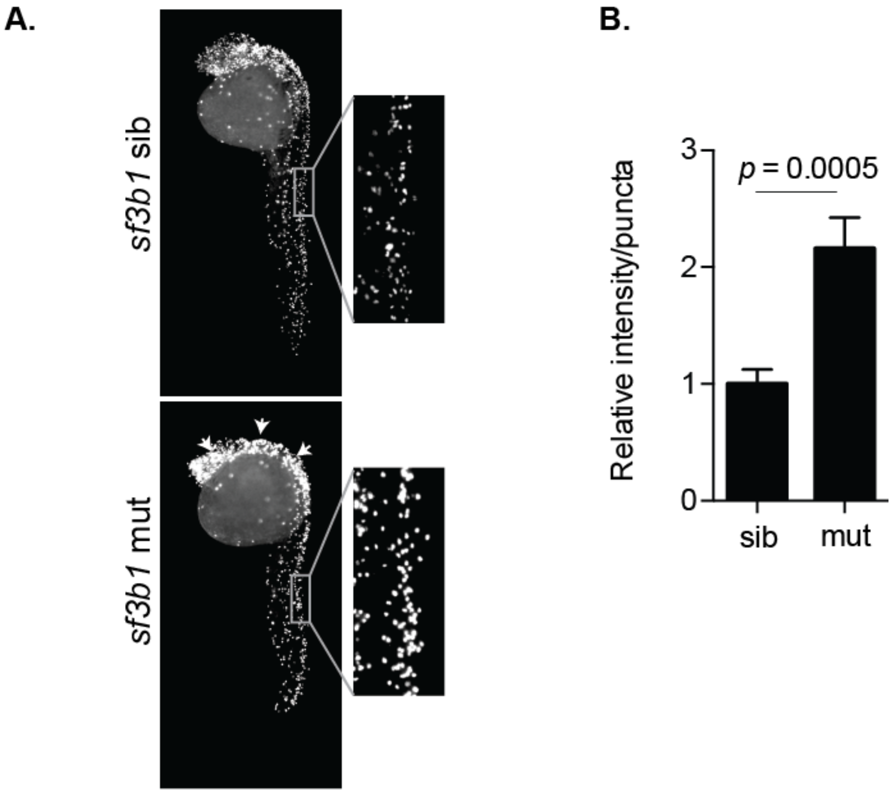

Figure S3

Figure S3. The splicing factor mutant *sf3b1*^{hi3394} have elevated levels of H3S10P. (A) Embryos were analyzed at 31 hpf by whole-mount immunofluorescence to detect histone H3 phosphorylation at serine 10 (H3S10P). Arrowheads mark increased H3S10P staining in the head of the *sf3b1*^{hi3394aTg} mutants. Magnified representative images of neural tissue highlighting differences in staining. **(B)** Neural tissue from the tails of *sf3b1* ^{hi3394aTg} mutants and siblings was quantified for intensity of H3S10P staining and normalized to the average number of H3S10P positive puncta per sibling control.

Disease Models & Mechanisms • Supplementary information
